# Oviduct Infection and Hydrosalpinx in DBA1/j Mice Is Induced by Intracervical but Not Intravaginal Inoculation with *Chlamydia muridarum*

**DOI:** 10.1371/journal.pone.0071649

**Published:** 2013-08-05

**Authors:** Lingli Tang, Hongbo Zhang, Lei Lei, Siqi Gong, Zhiguang Zhou, Joel Baseman, Guangming Zhong

**Affiliations:** 1 Department of Microbiology and Immunology, University of Texas Health Science Center at San Antonio, San Antonio, Texas, United States of America; 2 Departments of Clinic Laboratory, Pathology and Endocrinology, Second Xiangya Hospital, Central South University, Changsha, Hunan, China; Oregon State University, United States of America

## Abstract

Intravaginal infection with *C. muridarum* in mice often results in hydrosalpinx similar to that found in women urogenitally infected with *C. trachomatis*, making the *C. muridarum* lower genital tract infection murine model suitable for studying *C. trachomatis* pathogenesis. To our surprise, DBA1/j mice were highly resistant to hydrosalpinx following an intravaginal infection with *C. muridarum* although these mice were as susceptible to lower genital tract infection as other mouse strains. A significantly lower level of *C. muridarum* organisms was recovered from the oviduct of DBA1/j mice, correlating the resistance to hydrosalpinx with reduced ascension of *C. muridarum* to the oviduct. The DBA1/j resistance to hydrosalpinx was effectively overcome by intracervical inoculation with *C. muridarum*. The intracervically inoculated DBA1/j mice developed severe hydrosalpinx with the highest levels of live *C. muridarum* organisms recovered from uterine tissue on day 3 and oviduct tissue on day 7 post inoculation while in intravaginally inoculated DBA1/j mice, the peak of live organism recovery from uterine tissue was delayed to day 7 with no rise in the amount of live organisms recovered from the oviduct. These observations have not only validated the correlation between hydrosalpinx and live organism invasion in the oviduct but also demonstrated that the intracervical inoculation, by promoting rapid chlamydial replication in the uterine epithelial cells and ascension to the oviduct of DBA1/j mice, may be used for further understanding chlamydial pathogenic mechanisms. The above findings also suggest that strategies aimed at reducing tubal infection may be most effective in blocking tubal pathology.

## Introduction

Lower genital tract infection with *Chlamydia trachomatis*, if untreated in time, can lead to pathology in the upper genital tract, potentially resulting in severe complications such as ectopic pregnancy and tubal factor infertility [1]. It is thought that the chlamydial intracellular infection-induced inflammatory responses may contribute significantly to the upper genital pathology [2,3]. However, it remains unclear how the chlamydial organisms ascend and trigger the tubal pathology-causing inflammation. The lack of knowledge on chlamydial pathogenesis has also hampered the development of prevention strategies, including no licensed anti-*C. trachomatis* vaccine.

*Chlamydia muridarum* organisms have been extensively used to study the mechanisms of *C. trachomatis* pathogenesis and immunity [4–6] although *these* organisms cause no known human diseases. This is because intravaginal inoculation of mice with *C. muridarum* can often lead to hydrosalpinx, which closely mimics the tubal pathology induced by *C. trachomatis* in humans. Careful examination of hydrosalpinx in mouse oviduct revealed that hydrosalpinx is caused by fibrotic blockage of the oviduct lumen [7]. Thus, hydrosalpinx has been proposed as a surrogate marker for tubal occlusion and tubal factor infertility [7–9]. However, the precise mechanisms on how hydrosalpinx is induced by the *C. muridarum* organisms remain unknown although both host innate and adaptive immunity components have been proposed to play significant roles in upper genital tract pathology [10,11]. The finding of TLR2-mediated signaling pathways in *C. muridarum*-induced upper genital tract pathology examined microscopically on day 35 after infection is especially important [12]. Although it remains unclear whether the TLR2-mediated signaling pathways are sufficient for *C. muridarum*-induction of the irreversible hydrosalpinx, this finding suggested that the availability of chlamydial ligands for TLR2 detection might be essential for the chlamydial activation of pathogenic inflammatory responses. Nevertheless, many questions remain unanswered regarding the location, duration and extent of the TLR2 signaling. For example, does the TLR2 detection have to take place in the oviduct in order to induce hydrosalpinx-causing inflammation? How long and how strong must the chlamydial stimulation have to be in order to convert the reversible pyosalpinx into irreversible hydrosalpinx?

To address the above questions and to further understand the chlamydial pathogenic mechanisms, we correlated the hydrosalpinx with the presence of *C. muridarum* organisms in the mouse genital tract tissues. In the current study, we found that upon intravaginal inoculation with *C. muridarum*, DBA1/j mice failed to develop oviduct hydrosalpinx although this strain of mice was as susceptible to the intravaginal infection as the C57BL/6j and SJL/J mice. The lack of hydrosalpinx correlated well with significantly reduced ascending of *C. muridarum* organisms into oviduct tissue of DBA1/j mice. In contrast, when the *C. muridarum* organisms were inoculated into the DBA1/j mice intracervically, the organisms ascended to oviduct rapidly and extensively and remained in the oviduct for extended periods of time, which might be responsible for the enhanced hydrosalpinx. Thus, extensive infection in the oviduct epithelial cells appears to be a major determinant for hydrosalpinx development, suggesting that strategies for reducing oviduct infection may be most effective for preventing oviduct pathology.

## Materials and Methods

### Ethics statement

This study was carried out in strict accordance with the recommendations in the Guide for the Care and Use of Laboratory Animals of the National Institutes of Health. The protocol was approved by the Committee on the Ethics of Laboratory Animal Experiments of the University of Texas Health Science Center at San Antonio. Since the chlamydial infection is self-limiting, mice experience minimal suffering after infection. At the conclusion of each experiment or each time point, mice were sacrificed using overdose isoflurane.

### 1. Chlamydial organisms and infection

The *C. muridarum* organisms (Nigg strain) (also known as the agent of mouse pneumonitis or MoPn) used in the current study were propagated in HeLa cells (human cervical carcinoma epithelial cells, ATCC cat# CCL2.1), purified, aliquoted and stored as described previously [3,13]. Female C57BL/6J (stock number 000664), SJL/J (000686) and DBA1/j (000670) were purchased at the age of 5 to 6 weeks old from Jackson Laboratories (Bar Harbor, Maine). Each mouse was inoculated intravaginally with 2 X 10^5^ IFUs of live *C. muridarum* organisms in 20µl of SPG (sucrose-phosphate-glutamate buffer) or intracervically with the same amount of organisms but in 3µl of SPG. For intravaginal inoculation, the inoculum was delivered into mouse vagina using a 200µl micropipette tip as described previously [3]. For intracervical inoculation, a Non-Surgical Embryo Transfer Device (NSET, cat# 60010, ParaTechs Corp., Lexington, KY) was used and the manufacturer’s instruction (http://www.paratechs.com/nset/) was followed. Briefly, after connecting the NSET device onto a GeneMate P10 micropipette (0.5-10 µl size, BioExpress, Kaysville, UT), the micropipette was used to take up 3µl inoculum solution and then carefully adjusted to a setting of 3.5µl to generate a small air bubble at the tip of NSET. A speculum was gently placed into the mouse vagina to open up the vagina. The NSET loaded with the 3µl inoculum was inserted into the speculum and through the cervix. The inoculum was delivered by pressing the pipette plunger completely. The NSET device was immediately and gently removed without releasing the pipette plunger. The speculum was finally removed. For both types of inoculation, five days prior to the inoculation, each mouse was injected subcutaneously with 2.5mg Depo-provera (Pharmacia Upjohn, Kalamazoo, MI) to synchronize estrus cycle and increase mouse susceptibility to chlamydial infection. For *in vitro* infection of HeLa cells, HeLa cells grown on coverslips in 24-well plates containing DMEM (GIBCO BRL, Rockville, MD) with 10% fetal calf serum (FCS; GIBCO BRL) at 37^0^C in an incubator supplied with 5% CO_2_ were inoculated with *C. muridarum* organisms as described previously [3,13]. The infected cultures were processed for immunofluorescence assay as described below.

### 2. Monitoring live *C. muridarum* organism recovery from swabs and genital tract tissue samples

To monitor live organism shedding, vaginal swabs were taken on different days after the intravaginal or intracervical infection. Each swab was suspended in 500µl of ice-cold SPG followed by vortexing with glass beads, and the released organisms were titrated on HeLa cell monolayers in duplicates as described previously [3]. To monitor ascending infection, the whole genital tract tissue was harvested sterilely from each mouse on different days after infection as indicated in individual experiments. Each tissue was cut into different segments including cervix, uterus, uterine horn (both sides; in some experiments the uterus and uterine horns were cut as a single segment) and oviducts/ovaries (both sides; in some experiments, the left and right were pooled as a single tissue sample). Each segment sample was homogenized in 300µl of SPG using a 2-ml mini tissue grinder (Fisher Scientific, Pittsburgh, PA). After a brief sonication, the released live organisms were titrated as described above. The total number of IFUs per swab/tissue was calculated based on the number of IFUs per field, number of fields per coverslip, dilution factors and inoculation and total sample volumes. An average was taken from the serially diluted and duplicate samples for any given swab/tissue. The calculated total number of IFUs/swab or tissue was converted into log_10_ and the log_10_ IFUs were used to calculate means and standard deviation for each group at each time point.

### 3. Quantitating *C. muridarum* genome copies in genital tract tissue samples using quantitative PCR

A portion of each sample (50µl out of 300µl) was subjected to DNA extraction with QIAamp ^®^DNA Mini kit-250 (QIAGEN, FREDERICK, MD) by following the manufacturer’s instruction. Each DNA prep was resuspended in 100µl of water, and 1µl of the DNA samples was aliquoted for qPCR reaction using the primers complementary to chlamydial 16S ribosomal RNA coding region, including a forward primer, 5-CGCCTGAGGAGTACACTCGC-3’, and a backward primer, 5-CCAACACCTCACGGCACGAG-3, for amplifying a 208-bp fragment and a probe primer 5-CACAAGCAGTGGAGCATGTGGTTTAA-3; all primers were synthesized by Integrated DNA Technologies (Coralville, Iowa) for real time detection. A plasmid standard containing the 208-bp fragment of the chlamydial 16S ribosomal gene was used for qPCR quantification. The PCR reaction was carried out in a total volume of 20µl in a CFX96 Touch Deep Well Real-Time PCR Detection System with iQ Supermix real-time PCR reagent (both from Bio-Rad, Hercules, CA). The PCR running conditions were: initial denaturation at 95° C for 3 min followed by 40 cycles of amplification at 95° C 15s and 60° C for 1min. The results were expressed as total number of genome copies per sample and plotted in log_10_.

### 4. Evaluating mouse genital tract tissue pathologies and histological scoring

Mice were sacrificed on different days post infection as indicated in individual experiments, and the mouse urogenital tract tissues were isolated. Before the tissues were removed from mouse body, an *in situ* gross examination was performed for evidence of oviduct hydrosalpinx or any other related abnormalities of oviducts. The severity of oviduct hydrosalpinx was scored based on the following criteria: no hydrosalpinx (0), hydrosalpinx detectable only under microscopic examination (1), hydrosalpinx clearly visible with naked eyes but the size is smaller than the ovary on the same side (2), equal to the ovary on the same side (3) or larger than the ovary on the same side (4). The excised tissues, after photographing, were fixed in 10% neutral formalin, embedded in paraffin and serially sectioned longitudinally (with 5 µm/each section). Efforts were made to include cervix, both uterine horns and oviducts as well as lumenal structures of each tissue in each section. The sections were stained with hematoxylin and eosin (H&E) as described elsewhere [7]. The H&E stained sections were assessed by a pathologist blinded to mouse treatment and scored for severity of inflammation and pathologies based on the modified schemes established previously [7,14]. The uterine horns and oviducts were scored separately (only the oviduct scores were used in the current study). Scoring for dilation of uterine horn or oviduct: 0, no significant dilatation; 1, mild dilation of a single cross section; 2, one to three dilated cross sections; 3, more than three dilated cross sections; and 4, confluent pronounced dilation. Scoring for chronic inflammatory cell infiltrates (at the chronic stage of infection, the infiltrates mainly contain mononuclear cells while at the acute stage, neutrophils dominate the infiltration): 0, no significant infiltration; 1, infiltration at a single focus; 2, infiltration at two to four foci; 3, infiltration at more than four foci; and 4, confluent infiltration. Scores assigned to individual mice were calculated into means ± standard errors for each group of animals.

### 5. Immunofluorescence assay for cell culture and tissue section samples

HeLa cells grown on coverslips with chlamydial infection were fixed and permeabilized for immunostaining as described previously [15,16]. Hoechst dye (blue, Sigma) was used to visualize nuclear DNA. For titrating IFUs from swab and tissue homogenate samples, a mouse anti-chlamydial LPS antibody (clone# MB5H9, unpublished observation) plus a goat anti-mouse IgG conjugated with Cy3 (red; Jackson ImmunoResearch Laboratories, Inc., West Grove, PA) were used to visualize chlamydial inclusions. For detecting *C. muridarum* inclusions in tissue sections, 3 sections were collected from each tissue block with an interval of 10 cuts between each section. After deparaffinization and antigen retrieval treatments, the tissue section slides were blocked with 10% goat serum (catalog number 200-6210; Life Technologies, Grand Island, NY) in PBS for 1 h at room temperature. A rabbit anti-*C. muridarum* antiserum diluted at 1/3000 in PBS containing 1% BSA (unpublished data) was applied to the slides followed by a Cy2-conjugated goat anti-rabbit IgG (green) (Jackson ImmunoResearch Laboratories, West Grove, PA) together with Hoechst dye (blue) (Sigma-Aldrich, St. Louis, MO). All immunofluorescence-labeled samples were observed under an Olympus AX-70 fluorescence microscope equipped with multiple filter sets (Olympus, Melville, NY). The chlamydial inclusions were counted from oviduct cross sections in each slide. The total number of inclusions from all 3 slides was assigned to each mouse and used for calculating the means plus/minus standard deviations for each group.

### 6. Statistical analyses

Kruskal Wallis was used to analyze the differences in IFUs and genome copies recovered from mouse swabs and tissues. The pathology score data were analyzed with Wilcoxon rank sum test. The Fisher’s Exact test was used to analyze category data including the % of mice with oviduct hydrosalpinx.

## Results

### 1. Intravaginal inoculation with *C. muridarum* fails to induce hydrosalpinx in DBA1/j mice

**Figure 1:**
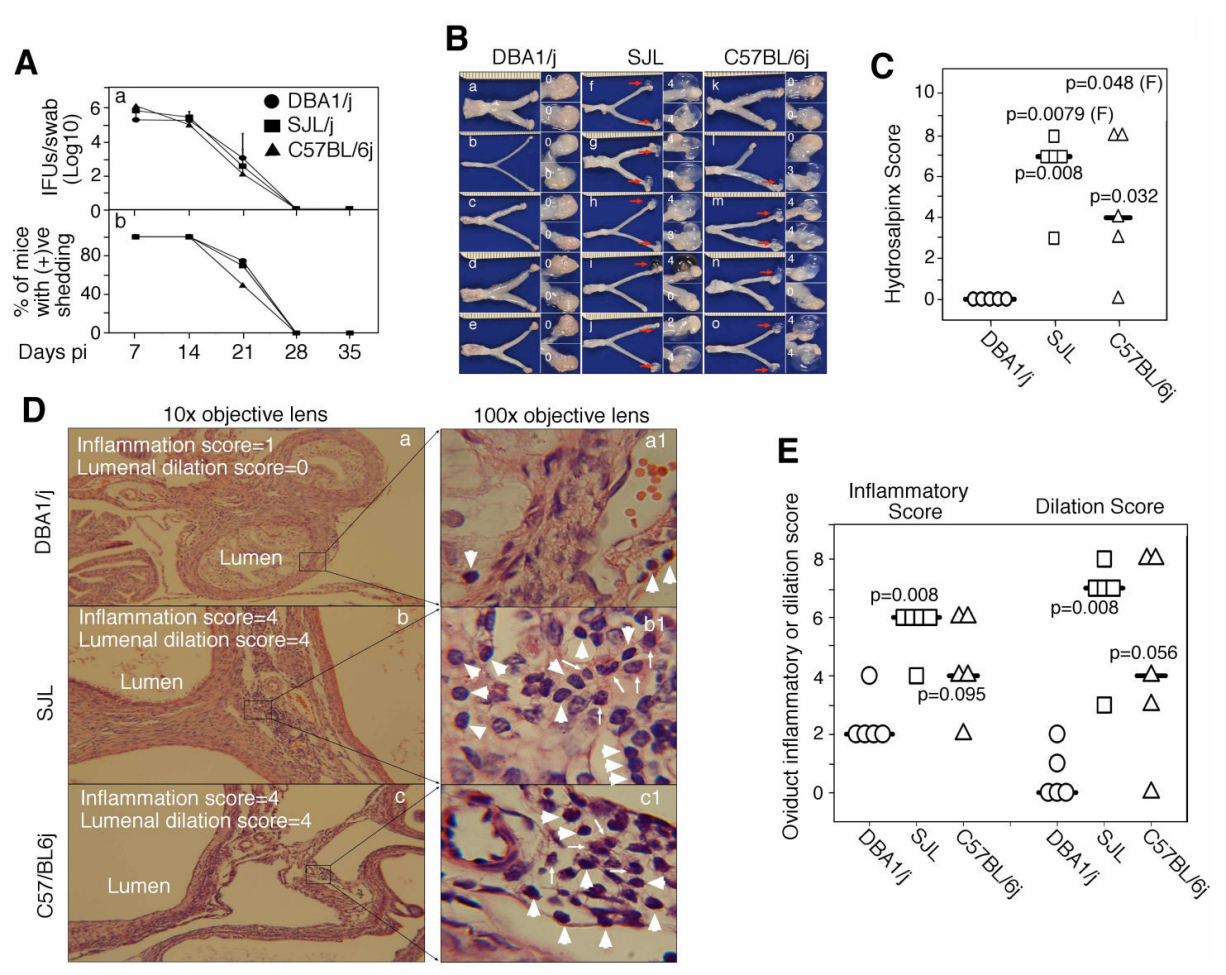
Live organism shedding and hydrosalpinx development in mice following an intravaginal infection with *C. muridarum*. (A) DBA1/j (filled circle, n=5), SJL/J (filled square, n=5) and C57BL/6j (filled triangle, n=5) female mice were intravaginally inoculated with *C. muridarum*. Live chlamydial organism shedding from the lower genital tract was monitored weekly post inoculation. Both the number of live organisms recovered from the vagina/cervix swabs expressed as log_10_ IFUs per swab (a) and percent of mice remaining positive in shedding live organisms (b) were calculated and displayed along the Y-axis. Similar shedding dynamics were observed in all 3 strains of mice. (B) Sixty days after infection, all mice were sacrificed for observing hydrosalpinx. The images of whole genital tract from each individual mouse were presented in the left columns with hydrosalpinx marked with red arrows and the amplified oviduct/ovary portion in the right columns with hydrosalpinx severity scores marked (see the materials and method section for hydrosalpinx gross pathology severity scoring criteria). (C) The hydrosalpinx severity scores were compared between DBA1/j and SJL/J or C57BL/6j mice. Both the hydrosalpinx incidence (Fisher’s Exact, or F) and severity (Wilcoxon Rank) were significantly higher in SJL/J or C57BL/6j mice than those of DBA1/j mice. (D) H&E-stained sections were made from the same genital tract tissues, and both inflammatory infiltration and lumenal dilation were scored for oviduct tissues. Representative images from each group of mice taken under 10X objective lens (left column with both inflammatory and lumenal scores marked, panels a to c) and 100X (right column, panels a1-c_1_) are shown. White arrowheads indicate mononuclear inflammatory cells while thin arrows indicate neutrophils. (E) The inflammatory and lumenal dilation scores were compared between DBA1/j and SJL/J or C57BL/6j mice (Wilcoxon Rank). SJL/J mice displayed significantly more severe inflammatory infiltration and lumenal dilation than the DBA/1j mice.

As shown in Figure 1, following an intravaginal infection with *C. muridarum* at 2 x 10^5^ IFUs, DBA1/j, C57BL/6j and SJL/J mice all displayed similar time courses of live organism shedding. The live organisms recovered from swabs of most mice on day 7 had a titer of more than 2 X 10^5^ IFUs, indicating that the intravaginally inoculated *C. muridarum* organisms were able to replicate and produce progeny infectious organisms in the genital tracts of all 3 strains of mice. The shedding remained positive for 3 weeks. By week 4, all mice ceased to shed live organisms, suggesting clearance of infection in the lower genital tract. These observations indicated that all mice were equally susceptible to lower genital tract infection with *C. muridarum*. When the mice were sacrificed on day 60 for observing genital tract pathology, we found that both C57BL/6j and SJL/J mice developed severe hydrosalpinx, a surrogate marker of oviduct blockage and infertility [7–9]. To our surprise, DBA1/j mice failed to do so (Figure 1B). Both the hydrosalpinx incidence and severity scores were significantly lower in DBA1/j than C57BL/6j and SJL/J mice (Figure 1C). The lack of hydrosalpinx in DBA1/j mice was further confirmed under microscopy (Figure 1D). Normal oviduct lumens were observed, and only scattered inflammatory cells could be detected in DBA1/j oviduct tissues. However, highly dilated oviduct lumens and extensive inflammatory infiltration were found in C57BL/6j and SJL/J mouse oviduct tissues. Most inflammatory cells appeared to be mononuclear cells although neutrophils were also detected, indicating chronic inflammation. The oviduct inflammatory and dilation scores were both significantly higher in C57BL/6j and SJL/J than DBA1/j mice (Figure 1E). The above results together have demonstrated that although all three strains of mice were equally susceptible to lower genital tract infection with *C. muridarum*, the DBA1/j mice failed to develop any significant hydrosalpinx.

### 2. The intravaginally inoculated *C. muridarum* organisms fail to effectively ascend to oviduct of DBA1/j mice

**Figure 2:**
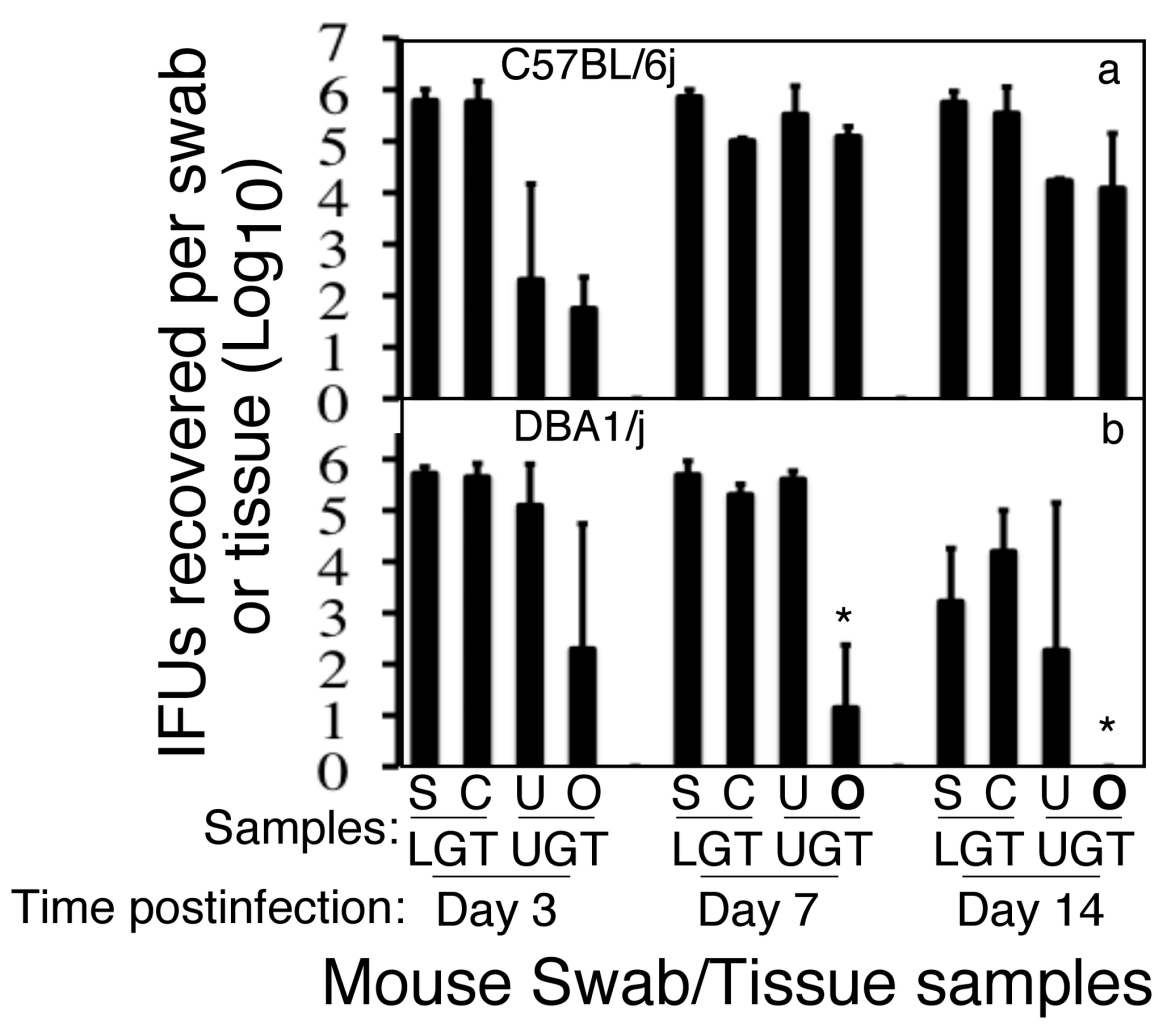
Live organism recovery from genital tract tissues of DBA1/j and C57BL/6j mice following intravaginal infection. C57BL/6j (panel a, n=15) or DBA1/j (b, n=15) mice were intravaginally inoculated with *C. muridarum* as described in Figure 1 legend and on days 3, 7 or 14 after inoculation as displayed along the X-axis, all mice were first swabbed (S) and then 5 mice from each group were sacrificed for harvesting the genital tract tissues at each time point. Each genital tissue was separated into cervix (C), uterus and uterine horns (U) and oviduct/ovary (O) for making homogenates. The live chlamydial organisms recovered from both the lower genital tract (LGT), including S & C, and upper genital tract (UGT), including U & O, tissues were titrated and the results were expressed as log_10_ IFUs per swab or tissue as displayed along the Y axis. The IFUs from the oviduct/ovary tissue homogenates were statistically significantly lower in DBA1/j comparing to those from C57BL/6j mice on both day 7 and 14 post inoculation (*p≤0.05).

To address why DBA1/j mice failed to develop hydrosalpinx, we monitored the live *C. muridarum* organism recovery from different sections of the genital tract tissues of both DBA1/j and C57BL/6j mice (Figure 2). In this set of experiments, besides collecting swabs (S), the genital tissues were also harvested from mice on days 3, 7 & 14 following an intravaginal infection with *C. muridarum*. Each genital tissue was separated into cervix (C), uterine and uterine horns (U) and oviducts/ovaries (O) for making homogenates, and live chlamydial organisms (expressed as IFUs) from the swab and separated tissue samples were titrated in cell culture (Figure 2). As expected, the numbers of live organisms recovered from the lower genital tract (LGT) samples, including the vaginal/cervical swabs and the cervix tissues, were no different between DBA1/j and C57BL/6j mice at any time points tested. When the IFUs recovered from the upper genital tract (LGT) samples were compared between CBA1/j and C57BL/6j mice, we found that the live organism recovery was significantly reduced in the oviduct and ovary samples of DBA1/j compared to those of C57BL/6j mice on both days 7 and 14 post inoculation although the uterus/uterine horn samples did not display any significant differences. Together with the pathology observations, we can conclude that the amount of chlamydial organisms in the oviduct may significantly impact hydrosalpinx development.

### 3. Intracervical inoculation with *C. muridarum* enhances oviduct infection in DBA1/j mice

**Figure 3:**
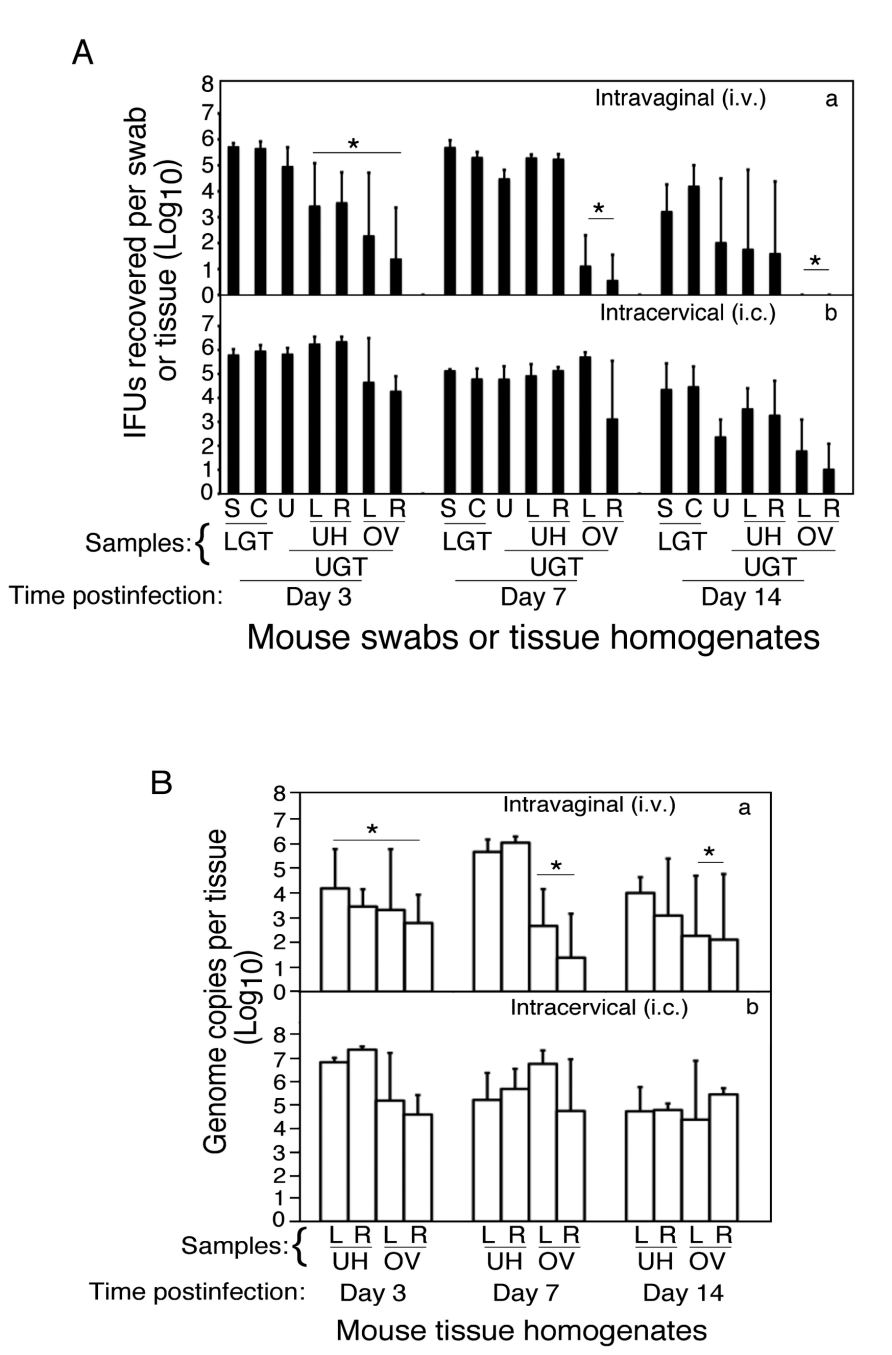
Live organism recovery from genital tract tissues of DBA1/j mice following intravaginal versus intracervical infection. (A) DBA1/j mice were inoculated with *C. muridarum* via either intravaginal (i.v., panel a, n=15) or intracervical (i.c., b, n=15) routes. On days 3, 7 or 14 after inoculation, all were first swabbed (S) and then 5 mice from each group were sacrificed for harvesting the genital tract tissues at each time point. Each genital tissue was separated into cervix (C), uterine (U), left (L) or right (R) uterine horns (UH) or oviduct/ovary (OV) for making homogenates. The live chlamydial organisms recovered from both the lower genital tract (LGT), including S & C, and upper genital tract (UGT), including U, UH(L), UH(R), OV(L) & OV(R), tissues as listed along the X-axis were titrated, and the results were expressed as log_10_ IFUs per swab or tissue as displayed along the Y axis. The IFUs from either the left or right oviduct/ovary tissue homogenates were statistically significantly higher in the intracervically infected compared to those of intravaginally infected DBA/1j mice on days 3, 7 and 14 post inocluation (*p≤0.05). (B) Both UH and OV tissue homogenate samples from mice described in Figure 3A legend above were subjected to quantitative PCR analyses and the numbers of genome copies (in log_10_) from each sample were calculated and displayed along the Y-axis. The intravaginally infected mice displayed significantly lower number of chlamydial genome copies recovered from the upper genital tract tissues than the intracervically infected mice (*p≤0.05).

To further strengthen the correlation between live organisms in the oviduct and hydrosalpinx, we evaluated whether increasing live organism infection in the oviduct of DBA1/j mice could induce these mice to develop hydrosalpinx. Intracervical inoculation has been shown to increase *C. trachomatis* organism ascending to the upper genital tract [17]. We then tested whether intracervical inoculation could also increase *C. muridarum* organism ascending into oviduct of DBA1/j mice. As shown in Figure 3A, live organism recovery from the lower genital tract (LGT) samples including swabs (S) and cervix (C) was not different between the intravaginally (i.v., panel a) and intracervically (i.c., b) inoculated DBA1/j mice. There were no significant differences in IFUs recovered from uterus (U) or left and right uterine horns (UH) either but with the exception of day 3 UH samples. However, when the IFUs from either left or right oviduct/ovary (OV) tissue samples were compared, we found that intracervical inoculation significantly increased the number of live organisms in these tissues on days 3, 7 & 14 post inoculation. This increase was validated by monitoring *C. muridarum* genome copies using quantitative PCR (Figure 3B). Similar to the live organism recovery, the intracervical inoculation resulted in significant increases in the number of *C. muridarum* genomes recovered from oviduct/ovary tissue samples at all time points tested. Thus, we have demonstrated that intracervical inoculation can enable *C. muridarum* organisms to significantly enter oviduct tissues of DBA1/j mice.

### 4. Intracervical inoculation with *C. muridarum* induces hydrosalpinx in DBA1/j mice

**Figure 4:**
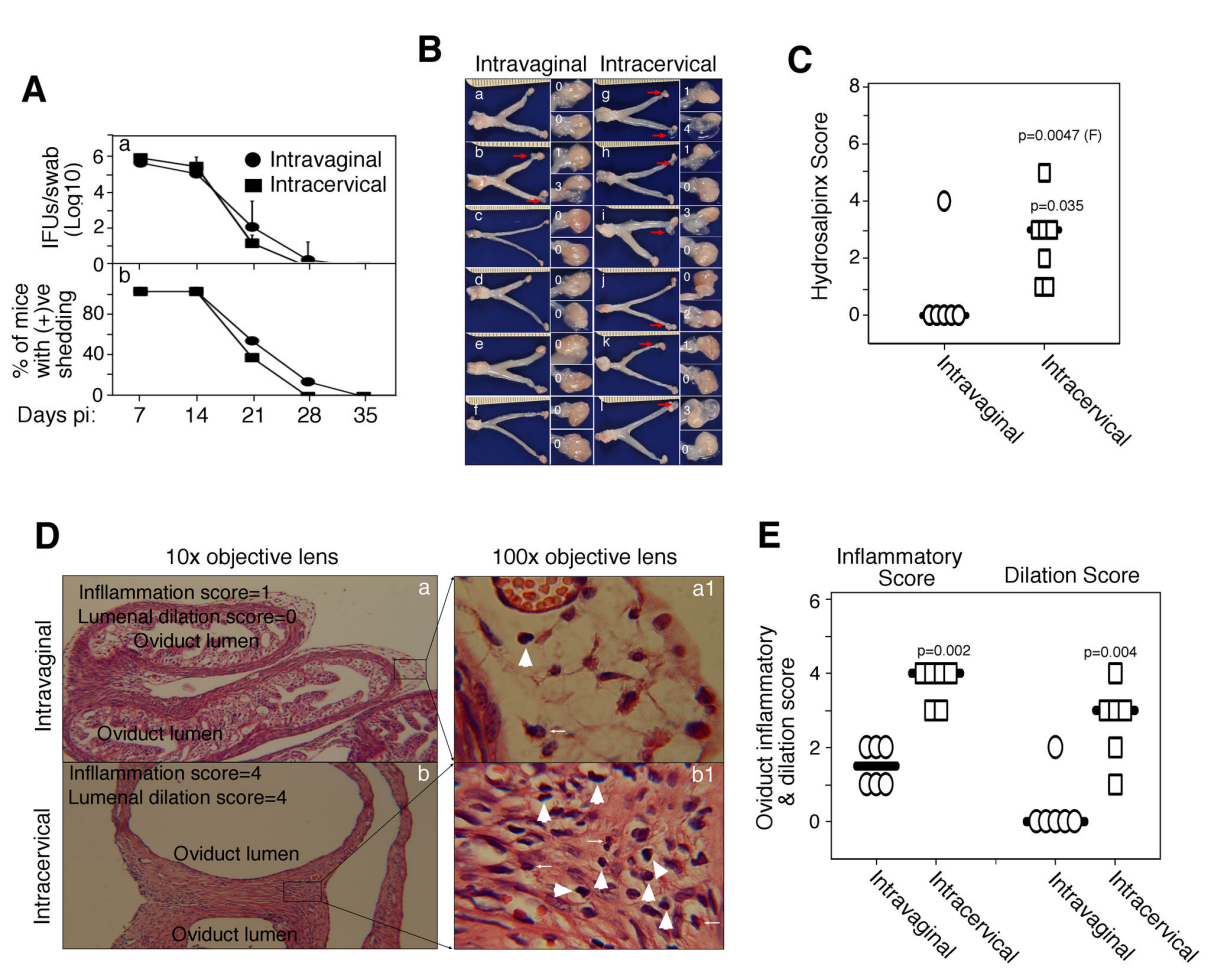
Hydrosalpinx in DBA1/j mice following intravaginal versus intracervical infections with *C. muridarum*. (**A**) DBA1/j mice were inoculated with *C. muridarum* via either intravaginal (filled circle, n=6) or intracervical (filled square, n=6) routes. The live organism shedding was monitored weekly, and both the amount of infectious organisms expressed as log_10_ IFUs (panel a) and percent of mice remained positive for shedding (b) were calculated and displayed along the Y-axis. Similar shedding courses were observed between these two groups. (**B**) Sixty days after inoculation, both groups of mice were sacrificed for observing hydrosalpinx as described in Figure 1B legend. (**C**) Both the incidence and severity of hydrosalpinx were compared between the intravaginal and intracervical groups as described in Figure 1C legend. Intracervical infection both significantly increased the incidence (p=0.0047, Fisher’s Exact or F) and enhanced the severity (p=0.035) of hydrosalpinx. (**D**) The genital tract tissues (after observing the gross pathology) were processed for microscopic observations of oviduct inflammatory infiltration and lumenal dilation under both 10X (panels a & b) and 100X (a1 & b1) as described in Figure 1D legend. (**E**) Both the oviduct inflammatory infiltration and lumenal dilation scores were compared between intravaginally and intracervically infected mice as described in the legend of Figure 1E. The intracervical infection significantly enhanced both inflammatory infiltration (p=0.002, Wilcoxon Rank) and lumenal dilation (p=0.004, Wilcoxon Rank) of oviduct in DBA/1j mice.

**Figure 5:**
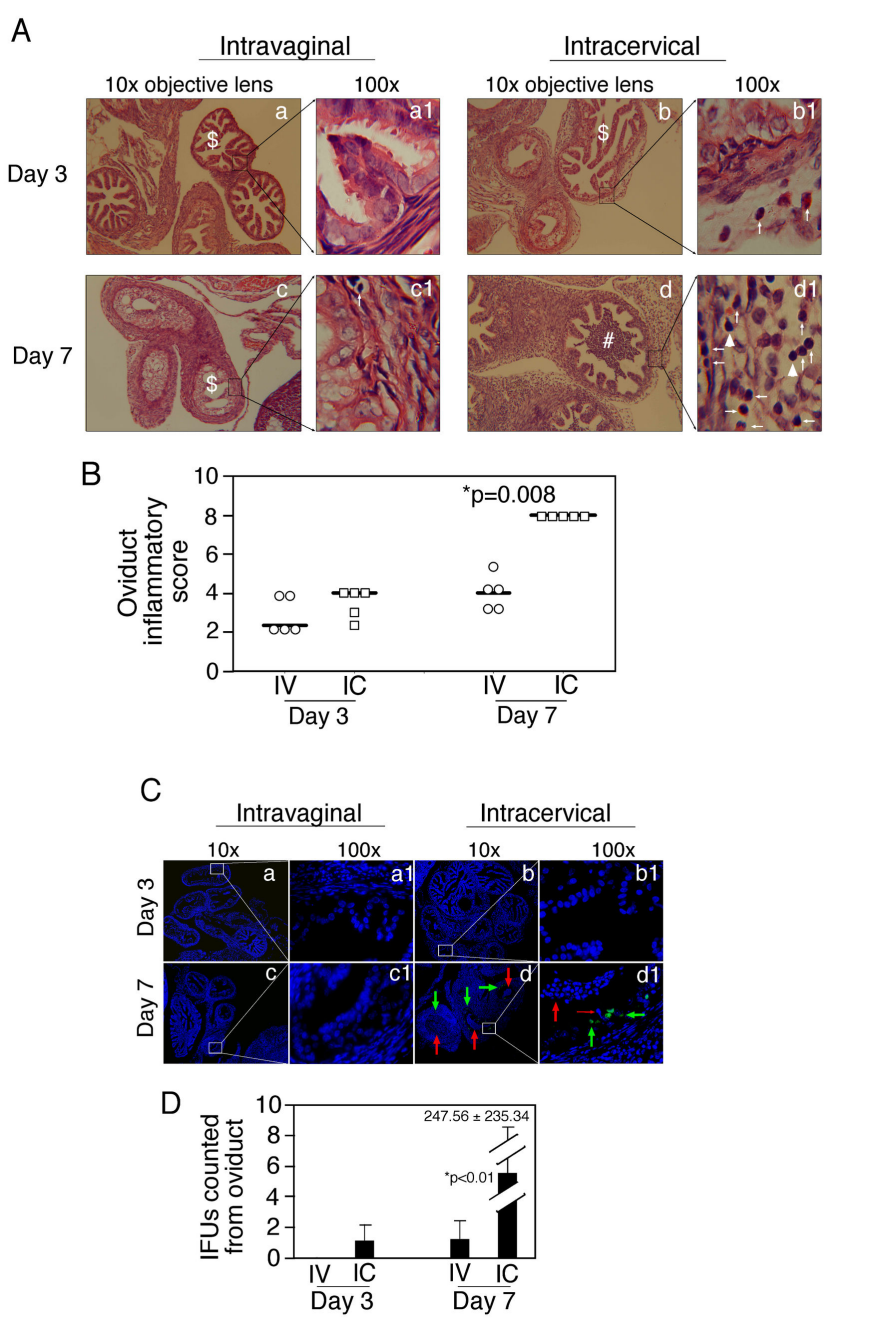
Acute inflammation and chlamydial inclusions in oviduct of DBA1/j mice infected with *C. muridarum* intravaginally vs. **intracervically**. (A) DBA1/j mice were inoculated with *C. muridarum* either intravaginally (panels a & c, n=10) or intracervically (panels b & d, n=10). On days 3 & 7 after inoculation, 5 mice from each group were sacrificed and the genital tract tissues were fixed for making sections. The H&E stained sections were examined for inflammation. Representative images taken under 10X (left columns) or 100X (right columns) objective lens from each group were shown. The intravaginally infected mouse oviduct sections appeared to be normal (white $ sign) without obvious lumenal dilation on either day 3 or 7, and only slight inflammatory infiltration was noted on day 7. However, obvious inflammatory infiltration was noted (panels b & b1, day 3) and severe lumenal inflammatory exudates (indicated with a white star, d & d1, day 7) were identified in intracervically infected mouse oviducts. White arrowheads indicate mononuclear inflammatory cells while thin arrows indicate neutrophils. The inflammatory infiltrates in the oviducts from each mouse were semi-quantitatively scored according to the criteria listed in the materials and methods section. The mean scores plus/minus standard deviations from each group were summarized in (B). Note that comparing to the intravaginal inoculation (IV), the intracervical inoculation (IC) significantly enhanced inflammatory infiltration (*p=0.008) in the oviducts 7 but not 3 days post-inoculation. (C) The tissue sections made as described above were also subjected to immunostaining of chlamydial antigens with a rabbit anti-*C. muridarum* antibody (green) and a Hoechst dye for DNA (blue). Obvious chlamydial inclusions were only detected in oviduct tissues of intracervically inoculated mice on day 7 after inoculation (panels d & d1). Thick red arrows point to inflammatory exudates in the oviduct lumens while thick green arrows point to chlamydial inclusions inside lumenal epithelial cells. The thin red arrow points to a neutrophil attacking a chlamydia-infected epithelial cell. (D) The chlamydial inclusions were counted from oviduct cross sections in each slide. The total number of inclusions from all 3 slides was assigned to each mouse and used for calculating the means plus/minus standard deviations for each group. Note that the number of chlamydial inclusions was significantly higher in the oviduct of mice inoculated intracervically (IC) than intravaginally (IV) on day 7 post inoculation.

Since intracervical inoculation of DBA1/j mice with *C. muridarum* significantly elevated the level of ascending infection in the oviduct, we further monitored the upper genital tract pathology (Fig. 4). DBA1/j mice after inoculation with *C. muridarum* either intracervically or intravaginally displayed similar time courses of live organism shedding from the lower genital tract (Figure 4A), which is consistent with the above observation. However, when mice were examined for genital tract pathology 60 days after inoculation, the intracervically inoculated mice developed obvious hydrosalpinx while the intravaginally infected mice only developed minimal levels of hydrosalpinx (Figure 4B). Both the incidence and severity of hydrosalpinx were significantly higher in intracervically than intravaginally inoculated mice. Intracervical inoculation both significantly increased the incidence and enhanced the severity of hydrosalpinx (Figure 4C). This gross pathology difference was confirmed under microscopy (Figure 4D). Both the oviduct inflammatory infiltration and lumenal dilation scores were statistically more severe in intracervically than intravaginally inoculated mice (Figure 4E). The oviduct chronic inflammation and hydrosalpinx observed on day 60 after infection in mice inoculated intracervically were further correlated with massive inflammatory cell infiltration (Figure 5A & B) and extensive epithelial cell infection (Figure 5C & D) on day 7 after inoculation in the oviducts of these mice.

## Discussion

Although chlamydial infection in the lower genital tract can lead to hydrosalpinx in the oviduct, it is not known whether chlamydial organism presence at the site where hydrosalpinx develops is necessary. In the current study, we have demonstrated that *C. muridarum* organisms have to reach the oviduct in substantial amounts in order to induce the development of hydrosalpinx. First, although C56BL/6j and SJL/J developed severe hydrosalpinx after an intravaginal inoculation, the DBA1/j mice failed to do so. This lack of hydrosalpinx correlated well with the reduced ascending infection in the oviduct. Second, the number of live organisms ascending to the uterus and uterine horns of DBA1/j mice was not different from that of C57BL/6j, suggesting that *C. muridarum* ascension into uterine tissues was not sufficient for inducing hydrosalpinx. Third, intracervical inoculation effectively increased the *C. muridarum* organisms ascending to the oviduct of DBA1/j mice. The increased infection in oviduct tissue resulted in hydrosalpinx. These observations have demonstrated that the lack of hydrosalpinx in the oviduct of intravaginally inoculated DBA1/j mice is not due to the inability of the mice to respond to the infection but due to insufficient ascending infection in the oviduct. Finally, we visualized the massive acute inflammatory cell exudates in the lumen of oviducts with extensive *C. muridarum* infection in the lumenal epithelial cells on day 7 after inoculation when DBA1/j mice were inoculated intracervically but not intravaginally.

In most strains of mice, intravaginal inoculation with *C. muridarum* organisms can lead to upper genital tract pathology. However, similar inoculation with *C. trachomatis* organisms often fails to do so. A recent study has shown that intracervical inoculation with *C. trachomatis* organisms can increase inflammatory pathology in the upper genital tract and stimulate robust host adaptive immune responses to chlamydial antigens [17]. This is probably due to the fact that intracervical inoculation allows the chlamydial organisms to bypass the cervix and directly access the uterine epithelial cells that may be more susceptible to chlamydial infection. We found that the DBA1/j mice were very resistant to upper genital pathology when intravaginally inoculated with *C. muridarum* organisms, and the resistance was effectively overcome by intracervical inoculation, suggesting that by bypassing the cervix, the *C. muridarum* organisms can gain significant access to the upper genital tract for causing pathology. However, careful analyses of the live organism recovery from the genital tract tissue segments revealed that even during intravaginal infection, significant numbers of live *C. muridarum* organisms were already present in the uterus and uterine horn tissues by day 7 after inoculation. There were no significant differences in either IFUs or genome copies recovered from uterus or uterine horn tissues between intracervically and intravaginally inoculated DBA1/j mice (Figure 3A). However, only the intracervically inoculated DBA/1j mice developed severe hydrosalpinx. These observations suggest that the increased uterine epithelial cell infection does not always lead to chlamydial ascension to oviduct and induction of hydrosalpinx. Indeed, the upper genital tract pathology induced by intracervically inoculated *C. trachomatis* organisms was mainly uterine horn dilation but not hydrosalpinx [17]. The question is what facilitated the ascension of the intracervically inoculated *C. muridarum* organisms to the oviduct and the induction of hydrosalpinx in the oviduct. The timing of chlamydial replication in the uterine epithelial cells might make the difference. By day 7, both intracervically and intravaginally inoculated DBA/1j mice displayed similarly high levels of live organisms in the uterus and uterine horn tissues. However, on day 3 after infection, the organism recovery from the intracervically infected mice was significantly higher than that of the intravaginally infected DBA1/j mice. It appeared that chlamydial infection in uterine epithelial cells peaked on day 3 after intracervical but day 7 after intravaginal inoculation (Figure 3). Maybe, in the intracervically infected mice, it was the early replication of *C. muridarum* organisms in the uterine epithelial cells that enabled the progeny organisms to ascend into oviduct. It might be possible that the progeny organisms produced early (during the first 3 days of infection) had a better chance to establish an infection in the oviduct and maintain a sustained inflammation leading to hydrosalpinx. However, by day 7, the adaptive immunity may have already occurred in the oviduct tissues. Thus, in the intravaginally infected mice, the progeny organisms produced in the uterine horn on day 7 might not be able to establish an infection in the oviduct due to immunity already established

The next question is how the *C. muridarum* organisms in the oviduct activate inflammatory responses that convert the reversible pyosalpinx into irreversible hydrosalpinx? It appears that host TLR2-mediated signaling pathways is involved since mice deficient in TLR2 failed to develop as robust a chronic inflammation as the wild type mice in the oviduct [12]. However, in this particular study, the chronic inflammation was only semi-quantitated under microscopy, and the gross pathology hydrosalpinx was not evaluated. Thus, it remains unclear whether TLR2-mediated signaling is sufficient for chlamydial induction of hydrosalpinx, the surrogate mark of tubal occlusion and infertility. In addition, mice deficient in MyD88, an essential adaptor of TLR2-mediated signaling pathways [18], developed even more severe hydrosalpinx [14]. Thus, the TLR2 signaling may not be essential for chlamydial induction of hydrosalpinx. As for the chlamydial ligands required for the activation of Chlamydia-induced inflammatory pathways, it was proposed that the cryptic plasmid might encode or regulate the ligands required for TLR2 signaling since wild type *C. muridarum* organisms activated TLR2 and induced upper genital tract pathology [12] while plasmid-free *C. muridarum* failed to do so [19,20]. Among the plasmid-encoded and regulated open reading frames [21], the plasmid-encoded Pgp3 might be the most promising candidate since it is not essential for plasmid replication [22] but abundantly produced during chlamydial infection in humans [23]. Pgp3 is also secreted into host cell cytosol [24] as stable trimers [25,26]. Most importantly, Pgp3 is able to stimulate macrophages to secrete inflammatory cytokines [24], which is consistent with a recent finding that the Pgp3 C-terminal domain resembles the trimer fold of TNFα [26]. The DBA1/j mouse model described in the current study may provide an alternative system for identifying novel chlamydial ligands and host pathways required for the development of hydrosalpinx.
